# Childhood lymphoma treatment impacts educational outcomes: a registry study from Sweden

**DOI:** 10.1007/s11764-022-01266-0

**Published:** 2022-11-04

**Authors:** Malin Lönnerblad, Reina Suominen, Arja Harila-Saari

**Affiliations:** 1 Department of Women’s and Children’s Health, Uppsala University, Uppsala, Sweden; 2 Department of Women’s and Children’s Health, Karolinska Institutet, Stockholm, Sweden; 3 Department of Special Education, Stockholm University, Stockholm, Sweden; 4 Department of Pediatric Oncology, Uppsala University Hospital, Uppsala, Sweden

**Keywords:** Lymphoma, Hodgkin lymphoma, Non-Hodgkin lymphoma, School grades, Post-compulsory education

## Abstract

**Purpose:**

This study aimed to explore educational outcomes in individuals diagnosed with lymphoma in childhood concerning school grade year 9 and attendance in high school and post-compulsory education. Whether sex or age at diagnosis affected the assessed variables was also explored.

**Methods:**

Data from 174 children born 1988–1996 and diagnosed with lymphoma before age 15 were matched with approximately five controls per patient. The mean time since diagnosis to receiving school year 9 grades was 4.88 years for Hodgkin lymphoma (HL) cases (mean age at diagnosis 10.62, 11.76, and 10.05 years for all, girls, and boys, respectively) and 7.79 years for non-Hodgkin lymphoma (NHL) cases (mean age at diagnosis 7.85, 7.87, and 7.84 years for all, girls, and boys, respectively).

**Results:**

We observed statistically significant differences between cases and controls in physical education, both for failing (*p* = 0.041) and the highest grade (*p* = 0.015). Compared with controls, HL cases were three times more likely to fail mathematics, and significantly fewer individuals in the whole lymphoma (*p* = 0.011) and NHL (*p* = 0.035) groups attended the third year of high school.

**Conclusions:**

Educational outcomes are impacted for children treated for lymphoma, especially in physical education. Since patients with HL are treated without central nervous system-directed therapy, other factors, such as absence from school, may affect school results. Physical late complications in lymphoma survivors warrant special attention.

**Implications for Cancer Survivors:**

The problems childhood lymphoma survivors face should be known by schools and parents, to enable their management. Children treated for lymphoma should be closely monitored and included in follow-up programs when needed, for example, to support physical activity.

## Introduction

There is a growing number of childhood lymphoma survivors, and the effects on cognition and academic performance following treatment and the cancer itself are gaining more interest. However, there is still a need for more information about educational outcomes, especially from the years in compulsory school, but also within the broader perspective of adult education. Lymphoma, including Hodgkin lymphoma (HL) and non-Hodgkin lymphoma (NHL), is the third most common form of cancer in children and adolescents [1]. HL occurs mostly in teenagers, and NHL presents in all pediatric age groups [1, 2]. The survival rate for childhood lymphoma is nowadays excellent [1–4], and there is a growing number of children attending school after being treated for lymphoma. HL is treated with combination chemotherapy usually including also glucocorticoids and, in patients with slow response, involved-field radiotherapy. Central nervous system (CNS) treatment is not part of the treatment for HL. NHL is a heterogeneous group of malignancies including Burkitt lymphoma, diffuse large B cell lymphoma, lymphoblastic lymphoma, and anaplastic large cell lymphoma [5–7]. Treatment is based on multiagent chemotherapy and, in contrast to HL, includes CNS-directed treatment in the form of intrathecal chemotherapy and in most protocols also high-dose methotrexate therapy. In rare cases with resistant CNS disease, cranial irradiation may be used. CNS treatment, mainly when radiotherapy is used but also with chemotherapy, has been associated with poorer neuropsychological performance especially in reaction speed, working memory, attention, and executive functions [8]. Cancer treatment causes absence from school and may lead to fatigue and psychosocial distress. These factors together may affect school performance during and after treatment.

### Lymphoma and educational outcomes

In a Finnish study by Lähteenmäki and colleagues from 2008, school results at the end of compulsory school in survivors of lymphoma and Wilms tumor born 1974–1986 were explored [8]. In that study, patients with NHL had lower school grade averages compared with controls. On the other hand, patients in the HL group had better results in some school subjects than their controls. A study by Andersen et al. investigated ninth grade school grades in Danish childhood cancer survivors born between 1982 and 1998 compared with controls who attended the same school. Their results showed that the academic performance in cancer survivors differed between cancer sites, and lymphoma was associated with lower-ranking grades [9].

Multiple studies have demonstrated the effects of childhood cancer on level of education and later employment, showing that children with non-CNS tumors have the same educational level as the general population [10–12]. Especially tumors in the CNS and cranial irradiation have been established to have a negative impact on cognitive functions leading to poorer academic performance and lower education level [10–17]. A Finnish registry study showed that childhood cancer survivors were less likely to have further education after comprehensive school but were not more likely to be unemployed [18].

Younger age at cancer diagnosis has been shown to be a risk factor for poorer school success in children with acute lymphoblastic leukemia and brain tumors [9, 12, 14, 19]. Moreover, it has been seen in previous studies that girls tend to suffer more from a cancer diagnosis in terms of school success than boys [8, 14, 15, 18], although girls tend to generally perform better in the Swedish school context compared with their male counterparts [20]. To the best of our knowledge, no national studies evaluating school grades in Swedish students diagnosed with lymphoma have previously been performed.

### Aim

The aim of this study was to explore educational outcomes in individuals born 1988–1996 in Sweden and diagnosed with HL and NHL in their childhood, in comparison with matched controls. Assessed variables included grades from school year 9, last year of compulsory school, and attendance in the third year of high school, as well as in municipal adult education, folk high school, and university. We also aimed to explore whether sex or age at diagnosis affected educational outcomes.

## Methods

### Setting

During the years for this study, Swedish children usually enrolled in preschool between the ages of 1 to 5 years old, and then most children [21] attended preschool class (*förskoleklass*), the year they turned 6 even though it was not part of the compulsory school until 2018. At the age of 7, they started compulsory school and usually finished the year they turned 16 and thereafter started high school. High school is not compulsory in Sweden, but the majority (> 90%) of students continue [22, 23]. Until 2012, a four-step scale was used to assess academic performance including fail (0 points) and the grades, pass (10 points), pass with distinction (15 points), and pass with special distinction (20 points). When the students apply for high schools, their grades would be converted into a merit rating, which is an average of the points for all grades. To qualify for high school up until 2011/2012, students had to have at minimum a pass as their final grade in Swedish, mathematics, and first foreign language (English). Since 2011/2012, the requirements to qualify for high school have become more demanding; students must have at least a pass as their final grade in eight or twelve school subjects according to which program they have chosen. For adolescents and adults who do not pass or attend high school, there is a possibility to attend municipal adult education (*Komvux*), where they may re-take failed courses or take courses or a whole program for high school. Sweden also has so-called folk high schools (*folkhögskola*) for students who wish to study the same courses as in high school or in the municipal adult education but in more accessible learning environment and often adapted to the students’ different needs [24, 25]. Many folk high schools also provide vocational or esthetic programs. For higher education, Sweden has both universities and university colleges, herein referred to collectively as universities, and the admission process is the same. High schools, municipal adult education, folk high schools, and universities are financed by taxes, and most often free of charge, and students can apply for study grants or loans when studying at least part-time.

### Participants

A total of 174 children treated for lymphoma were included. The distribution among the lymphoma cases was 63 children with HL and 111 children with NHL. Of children with NHL, 31 (17.8%) were treated for Burkitt lymphoma, 27 (15.5%) for B cell lymphoma, 21 (12.1%) for T cell lymphoma, 16 (9.2%) for anaplastic large cell lymphoma, 15 (8.6%) for pre-B cell lymphoma, and 1 (0.6%) of unknown NHL. Each child treated for lymphoma was matched with five controls by Statistics Sweden based on sex, birth year, and place of residence at the time of diagnosis, yielding 786 matched controls (Table 1). Controls had no history of cancer. Data about birth year, diagnosis, age at diagnosis, cancer treatment, relapses, and death year were obtained from the Swedish Childhood Cancer Registry, which has information about 94% of all children treated for cancer in Sweden [26]. Information from the years 2003–2017 about school grades, attendance in the third year of high school, and attendance in municipal adult education, folk high school, and university was obtained from Statistics Sweden [27]. The data did not include information about students attending special schools for children with intellectual disabilities. The number of children attending these schools per year during the years 2003–2012 was approximately 1.4% of all Swedish children [23], which is a small percentage, and thus, we find no reason to believe it would differ in the case and control groups, as most included cases have not had a treatment affecting the CNS. The number of students with missing data for unknown reasons, which could include attending a special school, was also similar in both groups (4.3% of cases and 5.1% of controls). From Statistics Sweden, we also received information about which course, Swedish or Swedish as a second language, they were enrolled in at school (Table 1) as well as data about the mother’s level of education, as it is well-known that a strong positive correlation exists between parental education and the child’s academic success [28, 29] with especially the mother’s education shown to be important [30, 31]. In our study, mother’s education was classified as low (school years 1–9 or less), medium (upper secondary school, which up until 1994, could be 2 or 3 years in Sweden for mainly vocational and theoretical educations, respectively), or high (higher education), and this information was available for all the cases and 99.5% of the controls.

**Table 1:** Characteristics of study participants diagnosed with Hodgkin lymphoma and non-Hodgkin lymphoma and their respective matched controls

	Cases *N* (%)	Controls *N* (%)	Chi-squared test, *p* value
Sex distribution			
All types of lymphoma	**174**	**786**	0.826
Girls	51 (29.3)	237 (30.2)	
Boys	123 (70.7)	549 (69.8)	
Hodgkin lymphoma	**63**	**288**	0.916
Girls	21 (33.3)	98 (34.0)	
Boys	42 (66.6)	190 (66.0)	
Non-Hodgkin lymphoma	**111**	**498**	0.851
Girls	30 (27.0)	139 (27.9)	
Boys	81 (73.0)	359 (72.1)	
Age at diagnosis			
All types of lymphoma			
0–5 years	40 (23.0)		
6–9 years	53 (30.5)		
10–14 years	81 (46.5)		
Hodgkin lymphoma			
0–5 years	8 (12.7)		
6–9 years	13 (20.6)		
10–14 years	42 (66.7)		
Non-Hodgkin lymphoma			
0–5 years	32 (28.8)		
6–9 years	40 (36.1)		
10–14 years	39 (35.1)		
Mother’s education ^a^			
All types of lymphoma			0.620
Low	21 (12.1)	77 (9.8)	
Medium	90 (51.7)	401 (51.0)	
High	63 (36.2)	304 (38.7)	
Missing		4 (0.5)	
Hodgkin lymphoma			0.576
Low	9 (14.3)	28 (9.7)	
Medium	33 (52.4)	157 (54.5)	
High	21 (33.3)	101 (35.1)	
Missing		2 (0.7)	
Non-Hodgkin lymphoma			0.828
Low	12 (10.8)	49 (9.8)	
Medium	57 (51.4)	244 (49.0)	
High	42 (37.8)	203 (40.8)	
Missing		2 (0.4)	

^a^ Low (school years 1–9 or less); medium (upper secondary school, which up until 1994, could be 2 or 3 years in Sweden for mainly vocational and theoretical educations, respectively); or high (higher education)

**Fig. 1:**
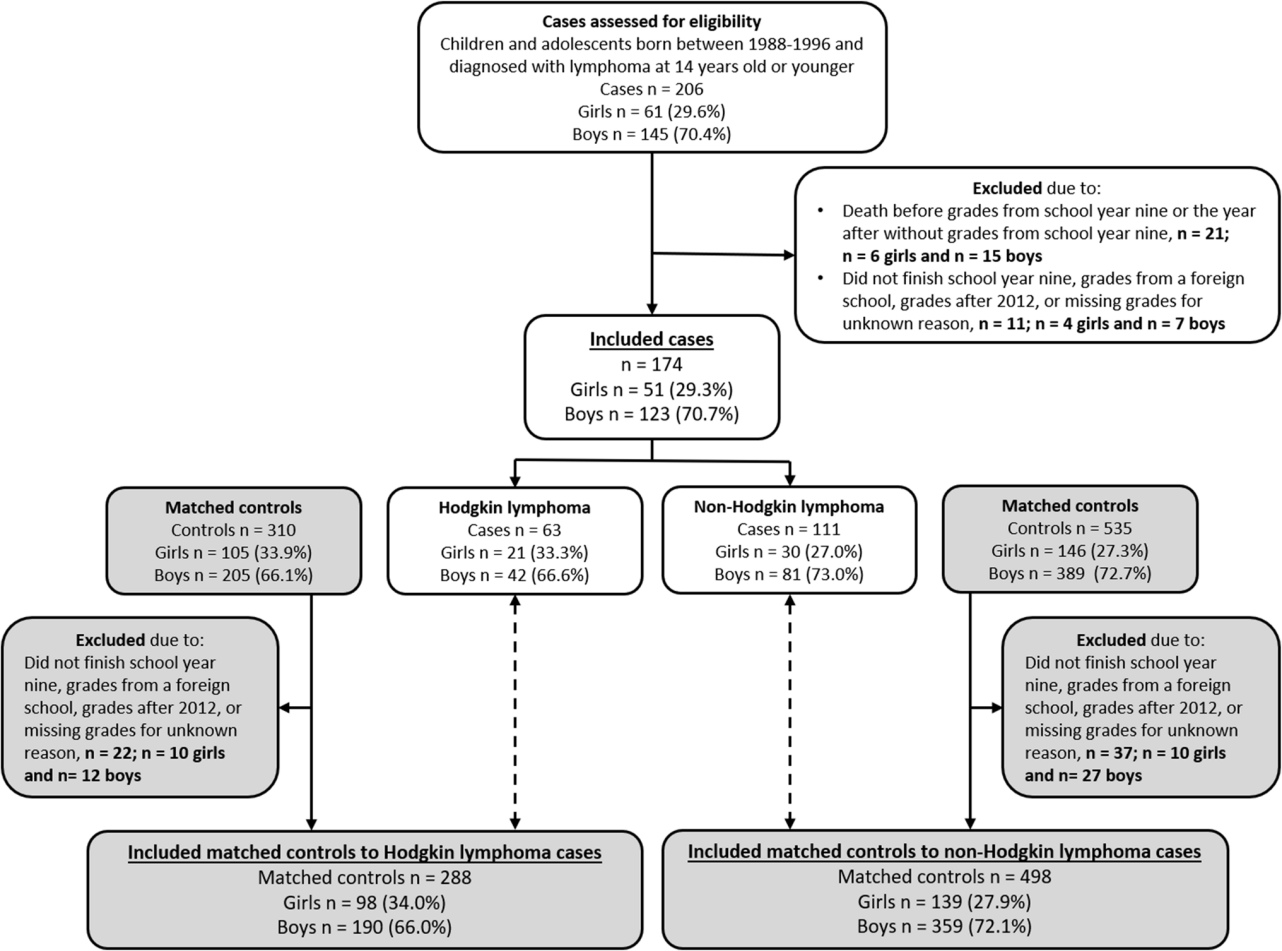
Inclusion and exclusion of cases (*n* = 174) and matched controls (*n* = 786). Cases are shown in white and controls in gray

The children treated for lymphoma were born between 1988 and 1996. This age group was selected because they would have received grades from school year 9, the last year of compulsory school, before the change in the grading system for compulsory school in 2013. The inclusion criterion consisted of being diagnosed with lymphoma before their fifteenth birthday, since the focus of this study was on the children who had already finished their treatment at the time they received their final grades. Children who passed away before receiving grades, did not finish school year nine, received grades from a foreign school, obtained grades after 2012 (due to a switch in the grading system in Sweden in 2013), or who were missing grades due to unknown reasons were excluded (Fig. 1).

### Ethics

This study was approved by the Regional Ethical Review Board in Stockholm (no. 2017/995–31/5). Parents provide written permission for their child to be included in the Swedish Childhood Cancer Registry. When patients turn 18, they are asked if they want to remain in the registry and informed that their data may be used in multiple studies without asking for their specific consent. All lymphoma patients were de-identified by Statistics Sweden, who performed the matching between cases and controls.

### Statistical methods

For this study, IBM SPSS version 28 was used for the statistical analyses. *P* values below 0.05 were considered to be statistically significant. Pearson’s Chi-squared test was used to determine whether there were differences in the background factors between cases for sex, mother’s level of education, and Swedish as first or second language. Logistic regressions were used to explore the differences between cases and controls for all the assessed variables, and all calculations were adjusted for mother’s education. When we explored results from school year 9, we used “fail” and the highest grade, “pass with special distinction,” as dependent variables. Only analyses of average grades were done with a *t* test. During the separate statistical analyses for the HL and NHL groups, matching was maintained so that they were compared with controls matching only the lymphoma cases in that group. When analyzing differences between children diagnosed at ages 0–5, 6–9, or 10–14 years, only cases were included. This was also the case when age at diagnosis and mean time since diagnosis were calculated. A *t* test was used to explore any significant differences for age at diagnosis between groups. When we calculated the mean time since diagnosis to receiving school year 9 grades, we estimated that all children received their final grades the first of June the year they got their grades and then subtracted each child’s time since diagnosis in days before converting to years.

## Results

### Background factors

No significant differences were found regarding sex (*p* = 0.826), mother’s level of education (*p* = 0.605), or Swedish as first or second language (*p* = 0.647) in the whole lymphoma group (Table 1). Background factors were also similar in children with previous HL and their controls in terms of sex (*p* = 0.916), mother’s education (*p* = 0.570), or Swedish as first or second language (*p* = 0.703) (Table 1). Between NHL cases and their matched controls, there were no significant differences in sex (*p* = 0.851), mother’s education (*p* = 0.823), or Swedish as first or second language (*p* = 0.293) (Table 1). The mean age at diagnosis was expectedly higher at 10.62 ± 3.35 years for children with HL and 7.85 ± 3.59 years for children with NHL, and the difference was significant (*p* = 0.001). The mean age at diagnosis for all girls treated for lymphoma was 9.47 ± 3.73, and for all boys treated for lymphoma, it was 8.59 ± 3.74; thus, the mean age for girls was slightly higher, but the difference was not significant (*p* = 0.161). For only HL cases, the mean age at diagnosis was 10.62, 11.76, and 10.05 years for all, girls, and boys, respectively, and for only NHL cases, the mean age at diagnosis was 7.85, 7.87, and 7.84 years for all, girls, and boys, respectively.

### School grades

In physical education, there was a statistically significant difference between all lymphoma cases and all controls, where the children treated for lymphoma were almost twice as likely to fail (odds ratio (OR), 1.86; confidence interval (CI), 1.03–3.38; *p* = 0.041). They were also less likely to obtain the highest grade and pass with special distinction, in physical education (OR, 0.59; CI, 0.39–0.90; *p* = 0.015) compared with controls. There was no statistically significant difference in grades between the lymphoma cases and their controls for fail or pass with special distinction in the subjects of Swedish, English, or mathematics. No statistically significant differences in grades between female or male lymphoma cases compared with their controls, nor for age at diagnosis in any subject, were observed. The mean time since diagnosis to receiving school year 9 grades was 6.74 ± 3.92, 4.88 ± 3.44, and 7.79 ± 3.80 years for all lymphoma cases, HL cases, and NHL cases, respectively.

**Fig. 2:**
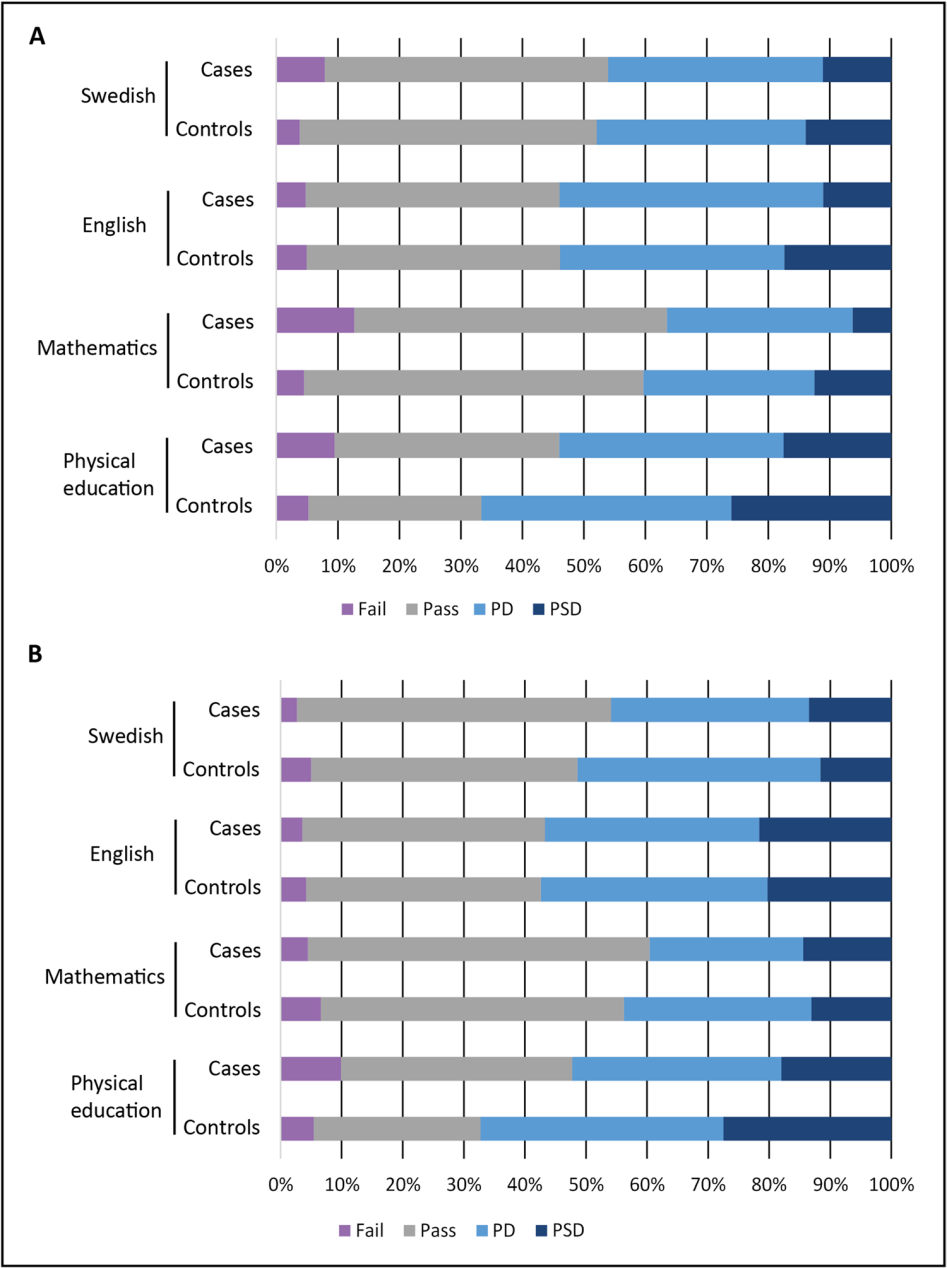
**A** Distribution (%) of all school grades for participants diagnosed with Hodgkin lymphoma and their matched controls as well as **B** those diagnosed with non-Hodgkin lymphoma and their matched controls. PD, pass with distinction. PSD, pass with special distinction

Between the children in the HL group and their controls, it was seen that cases were three times more likely to fail mathematics (OR, 3.0; CI, 1.16–7.76; *p* = 0.024) compared with controls (Fig. 2A). There were no significant differences for the highest grade in mathematics or in the subjects of English, Swedish, or physical education. Between the children in the NHL group and their controls, it was seen that cases were less likely to achieve the highest grade in physical education (OR, 0.58; CI, 0.35–0.99; *p* = 0.044) than controls, but there were no significant differences for fail or for the grades, pass with distinction, or pass with special distinction, in English, Swedish, or mathematics (Fig. 2B).

When we analyzed the average grade for all school subjects, we found that it was lower for cases than controls, but the difference was not significant (197 vs. 206; *p* = 0.063). The same trend was seen when female cases were compared with their female controls (207 vs. 222; *p* = 0.083), male cases with their male controls (193 vs. 199; *p* = 0.268), the HL group compared with their controls (189 vs. 204; *p* = 0.074), and the NHL group compared with their controls (201 vs. 207; *p* = 0.337).

### High school and post-compulsory education

When data about attending high school in the third and last year were analyzed, we observed that there were significantly fewer in the whole lymphoma group (Table 2) and in the NHL group who attended the third year of high school compared with their controls. There were no significant differences between children in the HL group and their controls, nor when boys and girls were compared to their controls separately or between children in different age groups at the time of diagnosis.

**Table 2:** Numbers (*N*), percentages, odds ratio (OR), confidence interval (CI), and *p* value (*p*) for qualification for attending the third year of high school, municipal adult education, folk high school, or university. Significant results are marked with an asterisk (*). HL, Hodgkin lymphoma. NHL, non-Hodgkin lymphoma

Post-compulsory education	*N* (%)	OR; CI; *p*
High school		
Lymphomas vs. control group	149 (85.6) vs. 723 (92.0)	0.52 (0.32–0.86; *p* = 0.011)*
HL vs. controls	54 (85.7) vs. 264 (91.7)	0.53 (0.23–1.23; *p* = 0.137)
NHL vs. controls	95 (85.6) vs. 459 (92.2)	0.51 (0.27–0.96; *p* = 0.035)*
Female cases vs. female controls	45 (88.2) vs. 223 (91.4)	0.42 (0.15–1.21; *p* = 0.109)
Male cases vs. male controls	104 (84.6) vs. 500 (91.1)	0.55 (0.31–0.97; *p* = 0.041)*
Differences between age groups ^a^		*p* = 0.060
Municipal adult education		
Lymphomas vs. control group	43 (24.7) vs. 196 (24.9)	0.90 (0.67–1.43; *p* = 0.897)
HL vs. controls	14 (22.2) vs. 78 (27.1)	0.76 (0.40–1.45; *p* = 0.401)
NHL vs. controls	29 (26.1) vs. 118 (23.7)	1.13 (0.70–1.82; *p* = 0.608)
Female cases vs. female controls	12 (23.5) vs. 63 (26.6)	0.84 (0.41–1.71; *p* = 0.632)
Male cases vs. male controls	31 (25.2) vs. 133 (24.2)	1.04 (0.66–1.64; *p* = 0.853)
Differences between age groups ^a^		*p* = 0.942
Folk high school		
Lymphomas vs. control group	26 (14.9) vs. 73 (9.3)	1.72 (1.06–2.77; *p* = 0.029)*
HL vs. controls	9 (14.3) vs. 23 (8.0)	1.93 (0.84–4.41; *p* = 0.119)
NHL vs. controls	17 (15.3) vs. 50 (10.0)	1.64 (0.90–2.97; *p* = 0.106)
Female cases vs. female controls	12 (23.5) vs. 24 (10.1)	2.74 (1.26–5.96; *p* = 0.011)*
Male cases vs. male controls	14 (11.2) vs. 49 (8.9)	1.30 (0.69–2.44; *p* = 0.410)
Differences between age groups ^a^		*p* = 0.591
University		
Lymphomas vs. control group	68 (39.1) vs. 352 (44.8)	0.80 (0.57–1.14; *p* = 0.219)
HL vs. controls	22 (34.9) vs. 127 (44.1)	0.67 (0.37–1.21; *p* = 0.185)
NHL vs. controls	46 (41.4) vs. 225 (45.2)	0.88 (0.57–1.36; *p* = 0.575)
Female cases vs. female controls	27 (52.9) vs. 137 (57.8)	0.80 (0.43–1.52; *p* = 0.499)
Male cases vs. male controls	41 (33.3) vs. 215 (39.2)	0.80 (0.52–1.22; *p* = 0.299)
Differences between age groups ^a^		*p* = 0.235

^a ^Only lymphomas included

Regarding attending municipal adult education or university at any time between 2006 and 2017, no significant differences between any groups were observed (Table 2). For attending folk high school during the same years, significant differences between cases and controls were observed, with almost twice as many in the lymphoma group who had attended folk high school compared with controls (Table 2).

## Discussion

In this registry-based study on educational outcomes for children treated for lymphomas, the grades from school year 9 in physical education were statistically poorer than in their matched controls. Especially, the children diagnosed with NHL performed worse in physical education compared with controls. This could be due to adverse effects caused by the treatment, including, e.g., glucocorticosteroids, that may lead to osteonecrosis [32] or vincristine, which commonly causes vincristine-induced peripheral neuropathy (VIPN) [33]. The symptoms for VIPN include muscle weakness, neuropathic pain, and loss of reflexes, which may lead to permanent loss of motor function and thus is problematic for physical activity. It has also been previously seen that childhood cancer survivors have a lower level of physical activity and fitness [34] that has been connected with cancer-related fatigue [35]. A study from 2016 that investigated the incidence of osteonecrosis in children treated for HL with whole-body magnetic resonance imaging scans found that ten of 24 participants (41.7%) had developed osteonecrosis [36]. Osteonecrosis can lead to severe pain, destruction of joints, and loss of mobility that could limit the child’s ability to participate fully in physical education.

In previous studies, children with lymphoma have performed worse than their controls in terms of academic success and education level [8, 9, 37], which was not seen as clearly in this study. Although there was no statistically significant difference between cases and controls for average grade, there was a trend with cases having a lower average than controls especially in the HL group, who were also three times more likely to fail mathematics compared with controls. One explanation could be a lower school attendance and lack of support during school absence. HL is diagnosed commonly in the teenage years, often close to the end of the ninth grade when the grades are given. Since HL treatment does not require CNS-directed treatment, cognitive effects are not expected of HL therapy. Our results are in contrast with those from the Finnish study by Lähteenmäki and colleagues [8], who found that children treated for HL performed at the same level or even better than their controls. Finland has a well-established system for home-schooling [38], which is not the case in Sweden [39], and could mean that the Swedish HL group had received less educational support than their Finnish counterparts during their treatment since treatment often leads to a high absence from school. Furthermore, in the Finnish study, children treated for NHL had lower grades in all assessed subjects, which was not seen in the current study. The grading system is more precise with six steps in Finland, which may increase the power to detect small differences. When analyzing data about attendance in post-compulsory education, we found that fewer NHL patients attended high school compared with their controls. When analyzed by sex, this difference was significant among the boys, but not the girls, which could be due to the low number of girls included in the study.

There were no significant differences between cases and controls for attending municipal adult education or university during the years 2006–2017, whereas significant differences between cases and controls were observed regarding folk high school attendance. It is possible that folk high schools are chosen by individuals diagnosed with lymphoma in their childhood and who subsequently suffer from some kind of late complication, as folk high schools are well-known to provide an accessible learning environment [24, 25].

A younger age at diagnosis has previously been observed to negatively affect school success, especially in children with NHL as the younger brain is considered to be more sensitive to toxic effects of CNS-directed cancer treatment [40, 41]. This was not seen in this study, which may be due to low participant number. In comparison with other studies [42], we did not observe that girls performed worse than boys compared to their sex-matched controls; however, this may be due to the small number of girls compared to boys in our study population.

Limitations for the study were the small sample size, which was especially problematic when comparing the different age groups and while analyzing data in the HL group that consisted only of 63 cases. Additionally, although an exclusion criterion for controls was that they did not have a cancer diagnosis, whether they (or cases) were diagnosed with any other chronic illness is unknown. Matching each case with five controls aimed to reduce any potential effects of such unknowns. Another limitation is the Swedish grading system, which has only four steps and thus provides less granularity than the six-step Finnish grading system (from fail to excellent, 4–10). We also found that our study population had more than two times as many boys than girls. It is well-known that a larger number of boys than girls are affected by childhood cancer but not to this extent, and unfortunately, we have no explanation for these skewed numbers. It is possible that we would have seen larger statistical differences between groups if more girls had been included (at least for grades from year 9); as in our earlier studies, we have seen the largest differences between female cases and controls. However, patients were matched with controls by sex to mitigate any potential contributions of sex on educational outcomes.

Study strengths include having high-quality data from the registry that is very representative as almost all the Swedish childhood lymphoma cases from this period are included, as well as very low number of students with missing data for unknown reasons (4.3% in case group and 5.1% in the control group). As the registry gains more data, it would be of value to repeat this study with a bigger sample size to strengthen the statistical analyses. It could then also be possible to look at the different NHL subgroups, as they differ from each other in many ways and may impact educational outcome. Furthermore, larger international studies could be performed, since children with lymphoma are mainly treated according to the same (or similar) protocols across Europe and therefore may face some of the same challenges following treatment. Such future studies could also be used to evaluate the effects of new treatment protocols on cognition and academic performance in the future. To the best of our knowledge, this is the first national study evaluating school grades in Swedish students diagnosed with lymphoma during childhood.

## Conclusions

Our results show that educational outcomes are affected for children and adolescents diagnosed with HL or NHL, especially in physical education. Since patients with HL and NHL are treated according to different protocols, e.g., those with HL do not receive treatment targeting the CNS, other factors likely affect these outcomes, for example, absence from school or physical late complications. Interestingly, almost twice as many in the lymphoma group had attended folk high school compared with controls, a type of education well-known to provide an accessible learning environment [24, 25], which may indicate a need for support also in post-compulsory education. More research into the factors affecting educational outcomes, including larger studies, is warranted.

### Implications for cancer survivors

It is positive that the academic outcome seems to be quite good for most children treated for lymphoma in Sweden, although this study shows that it is of particular importance to be aware of the challenges that childhood lymphoma survivors might face in terms of physical activity. Furthermore, there is a number of students, albeit small, who do not perform well in the academic subjects. Thus, children and adolescents treated for lymphoma should be closely monitored. Schools and parents of childhood lymphoma survivors should have knowledge about which problems the children face and how they could be managed, for example, by supporting physical activity or implementing other interventions. Both schools and hospitals should be involved in this work. As suggested in the international harmonization guidelines and in the Swedish long-term follow-up program after childhood cancer [43, 44], it is important that also educational issues are raised during medical follow-ups and, when needed, an assessment performed by a special education teacher or other educational specialist. To implement relevant educational services, some children might benefit from school re-entry programs during compulsory school as different programs have been shown to improve the collaboration between schools, hospitals, and families and also the child’s academic achievements [45, 46].

## Data Availability

The datasets analyzed during the current study are available from the corresponding author on reasonable request.
